# Supplementary material for: Entomopathogenic Nematodes and Their Symbiotic Bacteria from the National Parks of Thailand and Larvicidal Property of Symbiotic Bacteria against Aedes aegypti and Culex quinquefasciatus
Source: Biology (Basel). 2022 Nov 13;11(11):1658. doi: 10.3390/biology11111658 (PMC9687835; doi:10.3390/biology11111658)
Supplement: Supplementary file 1 [file biology-11-01658-s001.zip › Table S1.pdf]

Table S1. BLASTN search of ITS (680 bp) for *Heterorhabditis* isolates from National Parks in Thailand.

Table S1. BLASTN search of ITS (680 bp) for *Heterorhabditis* isolates (n = 6) from Phu Phan National Park/PP, Sakhon Nakhon Province, northern eastern Thailand.

Code	Maximum identity to	BLASTN				
		Accession number	Total score	Query coverage	E value	Identity
ePP3.5_TH	*Heterorhabditis indica* isolate Hi.QF6	MW548117	1098	100%	0	99.67%
ePP4.1_TH	*Heterorhabditis indica* voucher HeM	MF618313	1099	100%	0	99.83%
ePP7.1_TH	*Heterorhabditis indica* isolate Hi.QF6	MW548117	1062	100%	0	98.51%
ePP10.2_TH	*Heterorhabditis indica* voucher HeM	MF618313	1096	100%	0	99.67%
ePP21.1_TH	*Heterorhabditis indica* isolate EPN16	KP970843	1105	100%	0	100%
ePP32.2_TH	*Heterorhabditis indica* voucher HeM	MF618313	1105	100%	0	100%

Table S1. BLASTN search of ITS (680 bp) for *Heterorhabditis* isolates (n = 4) from Kaeng Krachan National Park/KKC, Phetchaburi Province, western Thailand (Cont.).

Code	Maximum identity to	BLASTN				
		Accession number	Total score	Query coverage	E value	Identity
eKKC2.5_TH	*Heterorhabditis baujardi* voucher HeTD1	MF618319	1146	100%	0	100%
eKKC20.5_TH	*Heterorhabditis baujardi* voucher HeTD1	MF618319	1129	100%	0	99.52%
eKKC25.1_TH	*Heterorhabditis indica* isolate EPN15	KP970842	1098	99%	0	100%
eKKC31.2_TH	*Heterorhabditis indica* voucher HeM	MF618313	1105	100%	0	100%

Table S1. BLASTN search of ITS (680 bp) for *Heterorhabditis* isolates (n = 8) from Namtok Samlan National Park/NTSL Saraburi Province, central Thailand (Cont.).

EPN Code	Maximum identity to	BLASTN				
		Accession number	Total score	Query coverage	E value	Identity
eNTSL1.2_TH	*Heterorhabditis indica* voucher HeM	MF618313	1105	100%	0	100%
eNTSL6.5_TH	*Heterorhabditis* sp. SGmg3	FJ751864	983	100%	0	99.09%
eNTSL13.3_TH	*Heterorhabditis* sp. SGmg3	FJ751864	992	100%	0	99.45%
eNTSL15.4_TH	*Heterorhabditis* sp. SGmg3	FJ751864	931	100%	0	97.10%
eNTSL26.4_TH	*Heterorhabditis indica* voucher HeM	MF618313	1101	100%	0	99.83%
eNTSL28.3_TH	*Heterorhabditis indica* voucher HeM	MF618313	1105	100%	0	100%
eNTSL34.2_TH	*Heterorhabditis* sp. SGmg3	FJ751864	941	100%	0	97.65%
eNTSL45.5_TH	*Heterorhabditis* sp. SGmg3	FJ751864	992	100%	0	99.45%
