# Supplementary material for: Entomopathogenic Nematodes and Their Symbiotic Bacteria from the National Parks of Thailand and Larvicidal Property of Symbiotic Bacteria against Aedes aegypti and Culex quinquefasciatus
Source: Biology (Basel). 2022 Nov 13;11(11):1658. doi: 10.3390/biology11111658 (PMC9687835; doi:10.3390/biology11111658)
Supplement: Supplementary file 1 [file biology-11-01658-s001.zip › Table S2.pdf]

Table S2. BLASTN search of 28S rDNA (633 bp) for *Steinernema* isolates from National Parks in Thailand.

Table S2. BLASTN search of 28S rDNA (633 bp) for *Steinernema* isolates (n = 7) from Phu Phan National Park/PP, Sakhon Nakhon Province, northern eastern Thailand.

Code	Maximum identity to	BLASTN				
		Accession number	Total score	Query coverage	E value	Identity
ePP1.2_TH	*Steinernema minutum* strain MP10	GU647156	992	99%	0	99.45%
ePP11.1_TH	*Steinernema guangdongense* strain GDc339	AY170341	905	100%	0	97.06%
ePP11.3_TH	*Steinernema guangdongense* strain GDc339	AY170341	911	99%	0	97.24%
ePP11.4_TH	*Steinernema guangdongense* strain GDc339	AY170341	887	100%	0	97.69%
ePP12.3_TH	*Steinernema guangdongense* strain GDc339	AY170341	924	99%	0	97.43%
ePP12.4_TH	*Steinernema guangdongense* strain GDc339	AY170341	909	100%	0	97.23%
ePP39.5_TH	*Steinernema yirgalemense*	AY748450	771	100%	0	93.93%

Table S2. BLASTN search of 28S rDNA (633 bp) for *Steinernema* isolates (n = 4) from Namtok Samlan National Park/NTSL Saraburi Province, central Thailand (Cont.).

Code	Maximum identity to	BLASTN				
		Accession number	Total score	Query coverage	E value	Identity
eNTSL9.4_TH	*Steinernema minutum* strain MP10	GU647156	846	100%	0	95.86%
eNTSL21.4_TH	*Steinernema minutum* strain MP10	GU647156	1007	100%	0	99.82%
eNTSL31.4_TH	*Steinernema minutum* strain MP10	GU647156	970	100%	0	98.38%
eNTSL39.2_TH	*Steinernema minutum* strain MP10	GU647156	1003	100%	0	99.64%

Table S2. BLASTN search of 28S rDNA (633 bp) for *Steinernema* isolates (n = 4) from Kaeng Krachan National Park, Phetchaburi Province, western Thailand (Cont.).

Code	Maximum identity to	BLASTN				
		Accession number	Total score	Query coverage	E value	Identity
eKKC4.1_TH	*Steinernema surkhetense* isolate CS44	MH837096	1146	100%	0	100%
eKKC19.3_TH	*Steinernema surkhetense* isolate CS44	MH837096	1146	100%	0	100%
eKKC28.1_TH	*Steinernema longicaudum*	GU395644	1038	100%	0	96.65%
eKKC39.1_TH	*Steinernema surkhetense* isolate CS44	MH837096	1146	100%	0	100%
