# Supplementary material for: Entomopathogenic Nematodes and Their Symbiotic Bacteria from the National Parks of Thailand and Larvicidal Property of Symbiotic Bacteria against Aedes aegypti and Culex quinquefasciatus
Source: Biology (Basel). 2022 Nov 13;11(11):1658. doi: 10.3390/biology11111658 (PMC9687835; doi:10.3390/biology11111658)
Supplement: Supplementary file 1 [file biology-11-01658-s001.zip › Table S3.pdf]

Table S3. BLASTN search of recA (588 bp) for *Photorhabdus* isolates from National Parks in Thailand.

Table S3. BLASTN search of recA (588 bp) for *Photorhabdus* isolates (n = 12) from Namtok Samlan National Park/NTSL Saraburi Province, central Thailand.

Code	Maximum identity to	BLASTN				
		Accession number	Total score	Query coverage	E value	Identity
bNTSL3.1_TH	*Photorhabdus luminescens* subsp. *akhurstii* strain FRG04	FJ862005	1086	100%	0	100%
bNTSL19.1_TH	*Photorhabdus luminescens* subsp. *akhurstii* strain FRG04	FJ862005	1108	100%	0	99.32%
bNTSL23.2_TH	*Photorhabdus luminescens* subsp. *akhurstii* strain LB06	LN835348	1083	99%	0	100%
bNTSL24.4_TH	*Photorhabdus luminescens* subsp. *akhurstii* strain FRG04	FJ862005	1081	100%	0	99.83%
bNTSL24.5_TH	*Photorhabdus luminescens* subsp. *akhurstii* strain LB06	LN835348	1083	99%	0	100%
bNTSL25.3_TH	*Photorhabdus luminescens* subsp. *akhurstii* strain FRG04	FJ862005	1081	100%	0	99.83%
bNTSL26.3_TH	*Photorhabdus luminescens* subsp. *akhurstii* strain LB06	LN835348	1083	99%	0	100%
bNTSL28.3_TH	*Photorhabdus luminescens* subsp. *akhurstii* strain FRG04	FJ862005	1081	100%	0	99.83%
bNTSL28.4_TH	*Photorhabdus luminescens* subsp. *akhurstii* strain FRG04	FJ862005	1048	100%	0	98.81%
bNTSL29.4_TH	*Photorhabdus luminescens* subsp. *akhurstii* strain LB06	LN835348	1038	99%	0	98.63%
bNTSL41.3_TH	*Photorhabdus luminescens* subsp. *akhurstii* strain FRG04	FJ862005	1181	100%	0	99.83%
bNTSL46.1_TH	*Photorhabdus luminescens* subsp. *hainanensis* strain C8404	FJ862004	1048	100%	0	99.81%

Table S3. BLASTN search of recA (588 bp) for *Photorhabdus* isolates (n = 12) from Kaeng Krachan National Park/KKC, Phetchaburi Province, western Thailand (Cont.).

Code	Maximum identity to	BLASTN				
		Accession number	Total score	Query coverage	E value	Identity
bKKC2.5_TH	*Photorhabdus luminescens* subsp. *akhurstii* strain FRG04	FJ862005	998	100%	0	97.28%
bKKC9.4_TH	*Photorhabdus luminescens* subsp. *akhurstii* strain FRG04	FJ862005	1003	100%	0	97.45%
bKKC14.1_TH	*Photorhabdus luminescens* subsp. *akhurstii* strain FRG04	FJ862005	998	100%	0	97.28%
bKKC14.3_TH	*Photorhabdus luminescens* subsp. *akhurstii* strain FRG04	FJ862005	1003	100%	0	97.45%
bKKC17.2_TH	*Photorhabdus luminescens* subsp. *hainanensis* strain C8404	FJ862004	1026	100%	0	98.13%
bKKC20.5_TH	*Photorhabdus luminescens* subsp. *akhurstii* strain FRG04	FJ862005	998	100%	0	97.28%
bKKC24.4_TH	*Photorhabdus luminescens* subsp. *akhurstii* strain LB06	LN835348	1083	99%	0	100%
bKKC25.1_TH	*Photorhabdus luminescens* subsp. *akhurstii* strain LB06	LN835348	1083	99%	0	100%
bKKC25.3_TH	*Photorhabdus luminescens* subsp. *akhurstii* strain LB06	LN835348	1083	99%	0	100%
bKKC25.5_TH	*Photorhabdus luminescens* subsp. *akhurstii* strain FRG04	FJ862005	1003	100%	0	97.45%
bKKC28.4_TH	*Photorhabdus luminescens* subsp. *hainanensis* strain C8404	FJ862004	1014	100%	0	97.79%
bKKC31.2_TH	*Photorhabdus luminescens* subsp. *akhurstii* strain LB06	LN835348	1083	99%	0	100%

Table S3. BLASTN search of recA (588 bp) for *Photorhabdus* isolates (n = 17) from Phu Phan National Park/PP, Sakhon Nakhon Province, northern eastern Thailand (Cont.).

Code	Maximum identity to	BLASTN				
		Accession number	Total score	Query coverage	E value	Identity
bPP3.4_TH	*Photorhabdus luminescens* subsp. *akhurstii* strain FRG04	FJ862005	1186	100%	0	100%
bPP3.5_TH	*Photorhabdus luminescens* subsp. *hainanensis* strain C8404	FJ862004	1048	100%	0	98.81%
bPP4.1_TH	*Photorhabdus luminescens* subsp. *akhurstii* strain FRG04	FJ862005	1181	100%	0	99.83%
bPP5.4_TH	*Photorhabdus luminescens* subsp. *akhurstii* strain LB06	LN835348	1183	99%	0	100%
bPP7.1_TH	*Photorhabdus luminescens* subsp. *akhurstii* strain LB06	LN835348	1044	99%	0	98.81%
bPP8.1_TH	*Photorhabdus luminescens* subsp. *akhurstii* strain LB06	LN835348	1066	99%	0	99.49%
bPP9.3_TH	*Photorhabdus luminescens* subsp. *akhurstii* strain LB06	LN835348	1055	99%	0	99.15%
bPP9.4_TH	*Photorhabdus luminescens* subsp. *hainanensis* strain C8404	FJ862004	1086	100%	0	100%
bPP10.2_TH	*Photorhabdus luminescens* subsp. *akhurstii* strain LB06	LN835348	1055	99%	0	99.15%
bPP10.3_TH	*Photorhabdus luminescens* subsp. *akhurstii* strain FRG04	FJ862005	1159	100%	0	99.15%
bPP13.5_TH	*Photorhabdus luminescens* subsp. *hainanensis* strain C8404	FJ862004	1075	100%	0	99.66%
bPP14.2_TH	*Photorhabdus luminescens* subsp. *akhurstii* strain LB06	LN835348	1055	99%	0	99.15%

Table S3. BLASTN search of recA (588 bp) for *Photorhabdus* isolates (n = 17) from Phu Phan National Park/PP, Sakhon Nakhon Province, northern eastern Thailand (Cont.).

Code	Maximum identity to	BLASTN				
		Accession number	Total score	Query coverage	E value	Identity
bPP17.3_TH	*Photorhabdus luminescens* subsp. *hainanensis* strain C8404	FJ862004	1081	100%	0	99.83%
bPP19.5_TH	*Photorhabdus luminescens* subsp. *akhurstii* strain LB06	LN835348	1083	99%	0	100%
bPP21.1_TH	*Photorhabdus luminescens* subsp. *hainanensis* strain C8404	FJ862004	1081	100%	0	99.83%
bPP32.2_TH	*Photorhabdus luminescens* subsp. *hainanensis* strain C8404	FJ862004	1053	100%	0	98.98%
bPP33.1_TH	*Photorhabdus asymbiotica* subsp. *australis* strain 9802892	FJ862018	1075	100%	0	99.66%
