# Supplementary material for: Entomopathogenic Nematodes and Their Symbiotic Bacteria from the National Parks of Thailand and Larvicidal Property of Symbiotic Bacteria against Aedes aegypti and Culex quinquefasciatus
Source: Biology (Basel). 2022 Nov 13;11(11):1658. doi: 10.3390/biology11111658 (PMC9687835; doi:10.3390/biology11111658)
Supplement: Supplementary file 1 [file biology-11-01658-s001.zip › Table S4.pdf]

Table S4. BLASTN search of recA (588 bp) for *Xenorhabdus* isolates from National Parks in Thailand.

Table S4. BLASTN search of recA (588 bp) for *Xenorhabdus* isolates (n = 2) from Namtok Samlan National Park/NTSL Saraburi Province, central Thailand (Cont.).

Code	Maximum identity to	BLASTN				
		Accession number	Total score	Query coverage	E value	Identity
bNTSL3.4_TH	*Xenorhabdus stockiae* strain TH01	FJ823425	1075	100%	0	99.66%
bNTSL30.5_TH	*Xenorhabdus indica* strain SabOM	FJ536262	1003	100%	0	97.45%

Table S4. BLASTN search of recA (588 bp) for *Xenorhabdus* isolates (n = 8) from Kaeng Krachan National Park/KKC, Phetchaburi Province, western Thailand (Cont.).

Code	Maximum identity to	BLASTN				
		Accession number	Total score	Query coverage	E value	Identity
bKKC11.1_TH	*Xenorhabdus stockiae* strain 858516	JX485977	976	100%	0	96.60%
bKKC12.2_TH	*Xenorhabdus stockiae* strain 858516	JX485977	981	100%	0	96.77%
bKKC13.5_TH	*Xenorhabdus stockiae* strain 858516	JX485977	976	100%	0	96.60%
bKKC19.1_TH	*Xenorhabdus stockiae* strain 858516	JX485977	976	100%	0	96.60%
bKKC19.5_TH	*Xenorhabdus stockiae* strain 858516	JX485977	981	100%	0	96.77%
bKKC21.1_TH	*Xenorhabdus stockiae* strain 858516	JX485977	981	100%	0	96.77%
bKKC32.3_TH	*Xenorhabdus stockiae* strain 858516	JX485977	977	100%	0	96.60%
bKKC38.3_TH	*Xenorhabdus stockiae* strain 858516	JX485977	981	100%	0	96.77%

Table S4. BLASTN search of recA (588 bp) for *Xenorhabdus* isolates (n = 8) from from Phu Phan National Park/PP, Sakhon Nakhon Province, northern eastern Thailand (Cont.).

Code	Maximum identity to	BLASTN				
		Accession number	Total score	Query coverage	E value	Identity
bPP6.3_TH	*Xenorhabdus indica* strain SabOM	FJ536262	1072	100%	0	99.83%
bPP11.1_TH	*Xenorhabdus ehlersii* strain DSM16337	FJ823398	950	99%	0	95.90%
bPP11.2_TH	*Xenorhabdus ehlersii* strain DSM16337	FJ823398	950	99%	0	95.90%
bPP11.3_TH	*Xenorhabdus ehlersii* strain DSM16337	FJ823398	950	99%	0	95.90%
bPP11.4_TH	*Xenorhabdus griffiniae* strain ID10	FJ823399	1081	100%	0	99.83%
bPP12.2_TH	*Xenorhabdus ehlersii* strain DSM16337	FJ823398	988	99%	0	96%
bPP12.3_TH	*Xenorhabdus ehlersii* strain DSM16337	FJ823398	950	99%	0	95.90%
bPP39.5_TH	*Xenorhabdus stockiae* strain 858516	JX485977	1003	100%	0	97.45%

Table S4. BLASTN search of recA (588 bp) for *Xenorhabdus* isolates (n = 6) from Huai Nam Dang National Park, Chiang Mai Province, northern Thailand (Cont.).

Code	Maximum identity to	BLASTN				
		Accession number	Total score	Query coverage	E value	Identity
bHND18.1_TH	*Xenorhabdus japonica* strain DSM16522	FJ823400	1022	99%	0	98.12%
bHND19.2_TH	*Xenorhabdus hominickii* strain Sm1	FJ536268	1186	100%	0	100%
bHND20.2_TH	*Xenorhabdus japonica* strain DSM16522	FJ823400	985	99%	0	97.09%
bHND26.5_TH	*Xenorhabdus japonica* strain DSM16522	FJ823400	985	99%	0	97.09%
bHND28.2_TH	*Xenorhabdus japonica* strain DSM16522	FJ823400	985	99%	0	97.09%
bHND30.5_TH	*Xenorhabdus japonica* strain DSM16522	FJ823400	985	99%	0	97.09%
