# Supplementary material for: Entomopathogenic Nematodes and Their Symbiotic Bacteria from the National Parks of Thailand and Larvicidal Property of Symbiotic Bacteria against Aedes aegypti and Culex quinquefasciatus
Source: Biology (Basel). 2022 Nov 13;11(11):1658. doi: 10.3390/biology11111658 (PMC9687835; doi:10.3390/biology11111658)
Supplement: Supplementary file 1 [file biology-11-01658-s001.zip › Table S5.pdf]

Table S5. Mortality of *Aedes aegypti* and *Culex quinquefasciatus* after exposure to whole cell suspension of symbiotic bacteria.

Bacterial code	Cumulative mortality of *Aedes aegypti*				Cumulative mortality of *Culex quinquefasciatus*			
	**24 h**	**48 h**	**72 h**	**96 h**	**24 h**	**48 h**	**72 h**	**96 h**
Photorhabdus bKKC20.5_TH	6.67± 9.62	10.00±9.62	11.11±3.33	12.22±0.00	21.11±15.03	45.56±21.17	60.00±12.62	61.11±1.92
Photorhabdus bKKC25.3_TH	25.56±6.67	34.44±3.33	36.67±1.92	36.67±1.92	17.78±1.92	50.00±6.94	68.89±6.94	83.33±1.92
Photorhabdus bPP3.5_TH	22.22±5.09	26.67±5.78	28.89±8.39	30.00±0.00	56.67±3.33	91.11±1.92	93.33±3.85	96.67±3.33
Photorhabdus bPP7.1_TH	34.44±9.62	47.78±5.09	48.89±6.94	48.89±0.00	33.33±11.55	63.33±29.06	80.00±12.02	85.56±1.92
Xenorhabdus bHND30.5_TH	10.00±17.32	22.22±18.36	22.22±0.00	22.22±0.00	7.78±3.85	11.11±3.33	15.56±5.09	24.44±6.94
Xenorhabdus bPP39.5_TH	34.44±53.99	35.56±1.92	38.89±5.78	38.89±0.00	2.22±3.85	11.11±10.18	16.67±3.85	17.78±1.92
Escherichia coli ATCC 25922	0.00±0.00	0.00±0.00	0.00±0.00	0.00±0.00	4.44±7.70	25.56±11.71	48.89±12.02	62.22±8.82
Distilled water	0.00±0.00	1.11±1.92	2.22±1.92	2.22±0.00	0.00±0.00	32.22±25.02	46.67±5.09	60.00±3.33
