# Supplementary material for: Entomopathogenic Nematodes and Their Symbiotic Bacteria from the National Parks of Thailand and Larvicidal Property of Symbiotic Bacteria against Aedes aegypti and Culex quinquefasciatus
Source: Biology (Basel). 2022 Nov 13;11(11):1658. doi: 10.3390/biology11111658 (PMC9687835; doi:10.3390/biology11111658)
Supplement: Supplementary file 1 [file biology-11-01658-s001.zip › Table S6.pdf]

Table S6. The *P*-values for bacteria tested against *Aedes aegypti* larvae showing significant differences compared to control groups at *P*-value ≤ 0.05.

	PP39.5	PP3.5	PP7.1	KKC20.5	KKC25.3	*E. coli*	DW
HND30.5	0.0105	0.0039	0.8522	0.9179	0.0795	0.0000	0.0000
PP39.5		0.7683	0.0072	0.0086	0.0000	0.0000	0.0000
PP3.5			0.0021	0.0030	0.0000	0.0000	0.0000
PP7.1				0.9546	0.1127	0.0000	0.0001
KKC20.5					0.1053	0.0000	0.0001
KKC25.3						0.0006	0.0093
E. coli							0.1561

1 HND30.5 = *Xenorhabdus japonica*
2 PP39.5 = *Xenorhabdus indica*
3 PP3.5 = *Photorhabdus luminescens* subsp. *hainanensis*
4 PP7.1 = *Photorhabdus luminescens* subsp. *akhurstii*
5 KKC20.5 = *Photorhabdus luminescens* subsp. *akhurstii*
6 KKC25.3 = *Photorhabdus luminescens* subsp. *akhurstii*
7 E. coli = *Escherichia coli* ATCC 25922
8 DW = Distilled water
