# Supplementary material for: Entomopathogenic Nematodes and Their Symbiotic Bacteria from the National Parks of Thailand and Larvicidal Property of Symbiotic Bacteria against Aedes aegypti and Culex quinquefasciatus
Source: Biology (Basel). 2022 Nov 13;11(11):1658. doi: 10.3390/biology11111658 (PMC9687835; doi:10.3390/biology11111658)
Supplement: Supplementary file 1 [file biology-11-01658-s001.zip › Table S7.pdf]

Table S7. The *P*-values for bacteria tested against *Culex quinquefasciatus* larvae showing significant differences compared to control groups at *P*-value ≤ 0.05.

	PP39.5	PP3.5	PP7.1	KKC20.5	KKC25.3	*E. coli*	DW
HND30.5	0.2967	0.0000	0.0000	0.0000	0.0000	0.0000	0.0000
PP39.5		0.0000	0.0000	0.0000	0.0000	0.0000	0.0000
PP3.5			0.0000	0.0000	0.0000	0.0000	0.0000
PP7.1				0.0004	0.089	0.0000	0.0000
KKC20.5					0.0228	0.5318	0.1968
KKC25.3						0.0009	0.0001
E. coli							0.4499

1. HND30.5 = *Xenorhabdus japonica*
2. PP39.5 = *Xenorhabdus indica*
3. PP3.5 = *Photorhabdus luminescens* subsp. *hainanensis*
4. PP7.1 = *Photorhabdus luminescens* subsp. *akhurstii*
5. KKC20.5 = *Photorhabdus luminescens* subsp. *akhurstii*
6. KKC25.3 = *Photorhabdus luminescens* subsp. *akhurstii*
7. *E. coli* = *Escherichia coli* ATCC 25922
8. DW = Distilled water
